# A multicenter prospective cohort study to evaluate feasibility of radio-frequency identification surgical guidance for nonpalpable breast lesions: design and rationale of the RFID Localizer 1 Trial

**DOI:** 10.1186/s12885-022-09394-7

**Published:** 2022-03-22

**Authors:** Bianca M. den Dekker, Anke Christenhusz, Thijs van Dalen, Lisa M. Jongen, Margreet C. van der Schaaf, Anneriet E. Dassen, Ruud M. Pijnappel

**Affiliations:** 1 Department of Radiology, University Medical Center Utrecht, Utrecht University, Heidelberglaan 100, 3584 CX Utrecht, the Netherlands; 2 Department of Surgery, Medisch Spectrum Twente Enschede, University of Twente, Enschede, the Netherlands; 3 Department of Surgery, Diakonessenhuis Utrecht, Utrecht, the Netherlands; 4 Department of Radiology, Diakonessenhuis Utrecht, Utrecht, the Netherlands; 5 Department of Radiology, Medisch Spectrum Twente Enschede, Enschede, the Netherlands; 6 Dutch Expert Centre for Screening, Nijmegen, the Netherlands

**Keywords:** Breast cancer, Localization, Breast conserving surgery, Radiofrequency identification, RFID

## Abstract

**Background:**

Breast cancer screening and improving imaging techniques have led to an increase in the detection rate of early, nonpalpable breast cancers. For early breast cancer, breast conserving surgery is an effective and safe treatment. Accurate intraoperative lesion localization during breast conserving surgery is essential for adequate surgical margins while sparing surrounding healthy tissue to achieve optimal cosmesis. Preoperative wire localization and radioactive seed localization are accepted standard methods to guide surgical excision of nonpalpable breast lesions. However, these techniques present significant limitations. Radiofrequency identification (RFID) technology offers a new, nonradioactive method for localizing nonpalpable breast lesions in patients undergoing breast conserving surgery. This study aims to evaluate the feasibility of RFID surgical guidance for nonpalpable breast lesions.

**Methods:**

This multicenter prospective cohort study was approved by the Institutional Review Board of the University Medical Center Utrecht. Written informed consent is obtained from all participants. Women with nonpalpable, histologically proven in situ or invasive breast cancer, who can undergo breast conserving surgery with RFID localization are considered eligible for participation. An RFID tag is placed under ultrasound guidance, up to 30 days preoperatively. The surgeon localizes the RFID tag with a radiofrequency reader that provides audible and visual real-time surgical guidance.

The primary study outcome is the percentage of irradical excisions and reexcision rate, which will be compared to standards of the National Breast Cancer Organisation Netherlands (NABON)(≤ 15% irradical excisions of invasive carcinomas). Secondary outcomes include user acceptability/experiences, learning curve, duration and ease of the placement- and surgical procedure and adverse events.

**Discussion:**

This study evaluates the feasibility of RFID surgical guidance for nonpalpable breast lesions. Results may have implications for the future localization techniques in women with nonpalpable breast cancer undergoing breast conserving surgery.

**Trial registration:**

Netherlands National Trial Register, NL8019, registered on September 12^th^ 2019.

**Supplementary Information:**

The online version contains supplementary material available at 10.1186/s12885-022-09394-7.

## Background

Breast cancer screening and improving imaging techniques lead to an increase in the detection rate of early, nonpalpable breast cancers [1]. For early breast cancer, breast conserving surgery is an effective and safe treatment [2]. Accurate intraoperative lesion localization during breast conserving surgery is essential for adequate surgical margins while sparing surrounding healthy tissue to achieve optimal cosmesis.

Various lesion localization methods are currently used. Preoperative wire placement under image guidance was first described in 1965 by Dodd and has been the standard localization method since [3–5]. This technique requires careful logistical planning as the wire needs to be placed on the day of surgery, which can lead to significant workflow inefficiencies and operative delays. Furthermore, there is little time left for the surgeon to evaluate the imaging after wire placement and to communicate with the radiologist. The wire entry site may differ from the ideal surgical approach. Other disadvantages of wire localization are the risk of migration or dislodgement and patient anxiety caused by the wire protruding from the patient’s skin.

Radioactive I-125 seed localization was designed to overcome these limitations and has shown non-inferiority to wire localization in surgical outcomes including surgical margins, reexcision and reoperation rates, specimen size and cosmetic result [4, 6, 7]. However, the use of radioactive seeds is constrained by stringent nuclear regulatory issues. Obtaining and maintaining proper licensing and meticulous tracking of the radioactive seed is mandatory. These regulatory disadvantages have limited the widespread adoption of this technique and have prompted research and development of nonradioactive, non-wire localization methods [8–10].

Radiofrequency identification (RFID) technology may offer an alternative method for localizing nonpalpable breast lesion in patients undergoing breast surgery. The RFID system consists of a RFID tag and a handheld reader. The RFID tag contains a microchip with a unique identification number and an antenna that responds to the radiofrequency signal send by the reader. The tag alters and reemits the signal to the reader, which responds with an audio signal and displays the tag’s unique identification number and the distance to the nearest end of the tag. The audible and visual feedback provide the surgeon with real-time guidance during the excision procedure.

Although data on the use of RFID localization is limited, resulting from a proof-of-concept study [11], some small, single-center cohort studies [12–15], and one retrospective cohort analysis [16], all authors conclude that RFID tags are safe and effective in localizing nonpalpable breast lesions. Further clinical research in a prospective multicenter cohort is necessary to substantiate these first reports. Therefore, the RFID Localizer 1 Trial aims to evaluate the feasibility of RFID surgical guidance for nonpalpable breast lesions in a prospective multicenter cohort study.

## Methods and analysis

### Study design and setting

This prospective multicenter cohort study was designed to evaluate feasibility of RFID surgical guidance for nonpalpable breast lesions in two non-academic hospitals that acted as early adopters of RFID localization. The standard localization technique was wire-localizaton in one hospital and radioactive I-125 seed localization in the other. The primary study outcome is the percentage of irradical excisions and reexcision rate. Secondary outcomes include user acceptability/experiences, learning curve, duration and ease of the placement- and surgical procedure and adverse events.

### Study population and recruitment

Women, ≥ 18 years of age, with nonpalpable, histologically proven in situ or invasive breast cancer, who can undergo breast conserving surgery with RFID localization are considered eligible for participation (Table 1). All cancer subtypes may be included, provided that the lesion is visible on ultrasound imaging to enable RFID placement procedure under ultrasound guidance. Women with a lesion located deeper than 7 cm from the skin when lying supine cannot undergo RFID localization due to the maximum reach of the RFID reader of 7 cm. Women who are pregnant or lactating, women with multicentric breast cancer and women who are unable to understand and sign the study specific informed consent form after the nature of the study has been fully explained are excluded.

**Table 1:** RFID Localizer 1 Trial patient eligibility

**Inclusion criteria**
Female patient ≥ 18 years of age Patient has a nonpalpable histologically proven in situ or invasive breast cancer that is visible on ultrasound Patient is scheduled for breast conserving surgery
**Exclusion criteria**
Lesion depth > 7 cm in supine position Patient has multicentric breast cancer Patient is pregnant or lactating Patient is unable to understand and sign the study specific informed consent form after the nature of the study has been fully explained *Patient will undergo neoadjuvant treatment*^a^

^a^Participating centers with access to I-125 seed localization will not include patients undergoing neoadjuvant treatment because in these patients a single procedure using I-125 seed is preferred over two procedures (using a marker followed by RFID tag placement)

In participating centers with access to I-125 seed localization, patients undergoing neoadjuvant treatment will be excluded because in these patients a single procedure using I-125 seed is preferred over two procedures (using a marker during neoadjuvant treatment followed by preoperative RFID tag placement). Study participants are recruited from the surgical outpatient clinic of the participating hospitals. Eligibility criteria are assessed during multidisciplinary meetings. Patient characteristics, including breast cup size, are recorded. Written informed consent is obtained from all study participants.

### Study procedures (Fig. 1)

**Fig. 1:**
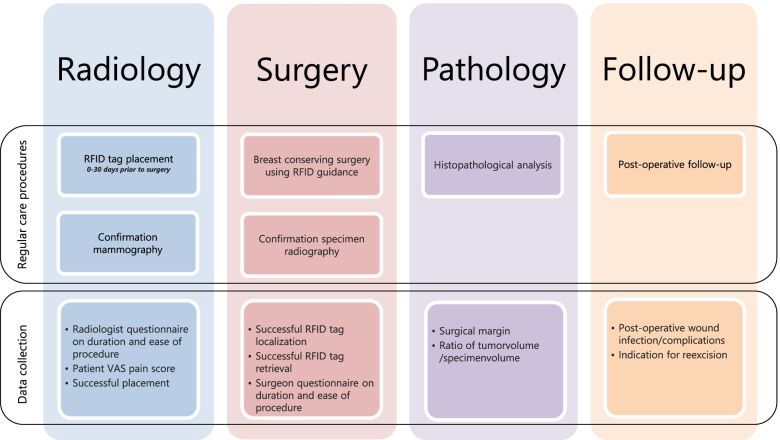
Overview of procedures and data collection of the RFID Localizer 1 Trial

Preoperative placement of an RFID tag is performed by an experienced breast radiologist, using ultrasound guidance, up to 30 days prior to surgery. The passive RFID tag is composed of a coil and a microchip that stores a unique identification number. The RFID tag is approximately 11 mm long and 2 mm in diameter. A polypropylene cap prevents migration in tissue. The RFID tag is inserted percutaneously through a small skin incision with a preloaded 12-gauge sterile needle applicator after injection of a local anaesthetic (lidocaine 2%). In tumors > 1 cm the RFID tag is placed intralesional, in lesions ≤ 1 cm the RFID tag is placed directly next to the tumor. The VAS pain score of the patient is recorded. A 2-view mammography (CC and MLO) is obtained to confirm correct position of the RFID tag. Successful placement is recorded, which is defined as 0-5 mm distance between any point of the tag to any point of the tumor measured on the post-placement imaging. Directly after the placement procedure the radiologist completes a questionnaire on the duration and ease of the procedure (Table 2).

**Table 2:** Questionnaires

Respondents specify their level of agreement or disagreement for the following statements on the five-level Likert scale: Strongly disagree, Disagree, Neither agree nor disagree, Agree, Strongly agree
**Radiologists**
The RFID tag applicator is easy to handle The RFID tag applicator needle is sharp enough to penetrate healthy breast tissue The RFID tag applicator needle is sharp enough to penetrate tumor tissue RFID tag deployment is simple The RFID tag is clearly visible on ultrasound I feel confident about the correct placement of the RFID tag
**Surgeons**
The RFID tag is easily identified pre-operatively using the loop probe The RFID tag is easily identified during surgery I feel confident that the RFID technology leads me to the correct location The RFID localization procedure is intuitive to use

**Fig. 2:**
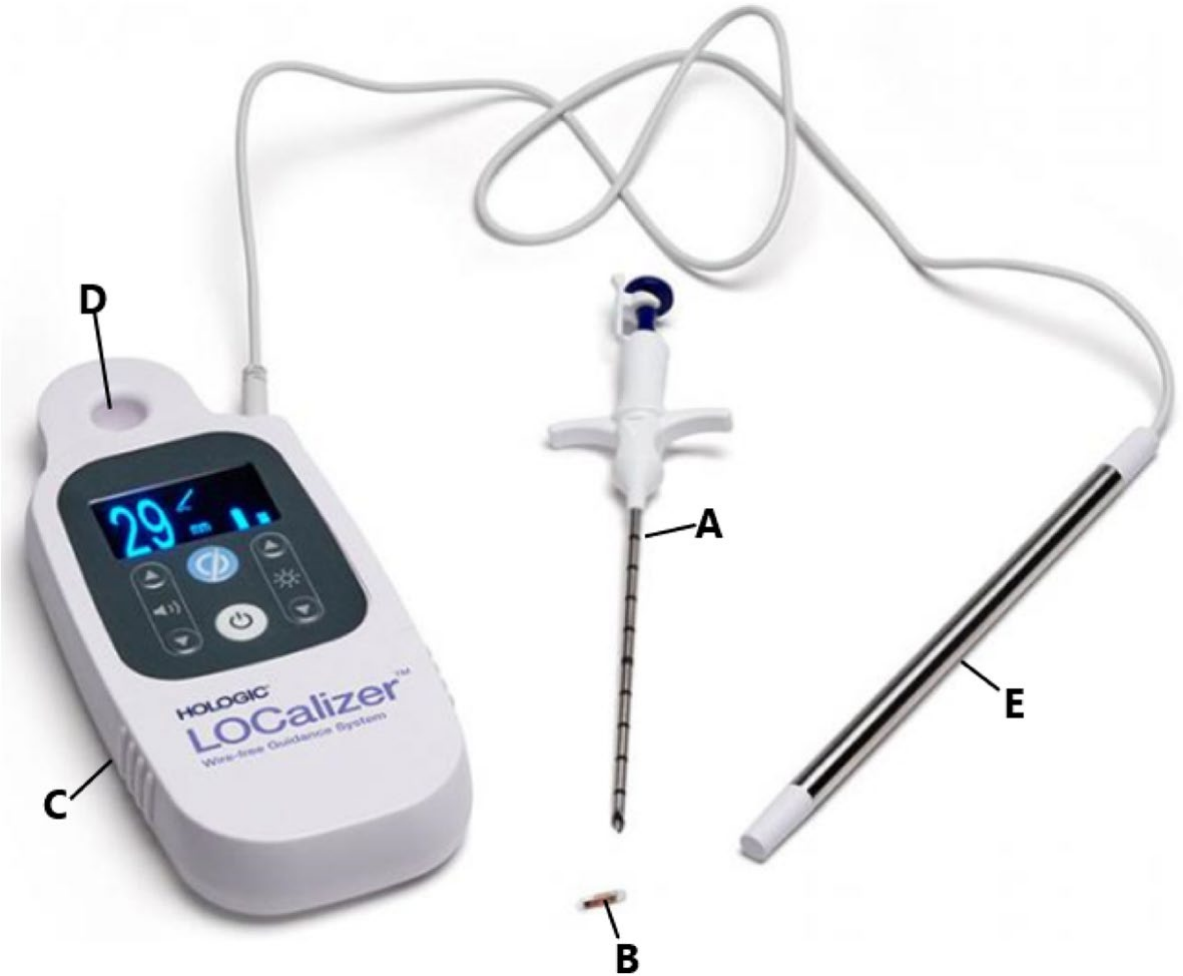
RFID LOCalizer system (Hologic). The RFID LOCalizer system (Hologic) consists of a preloaded 12-gauge sterile needle applicator (**A**) containing the RFID tag (**B**), a handheld, portable, battery-operated reader device (**C**) with a loop probe with a detection up to 7 cm of depth (**D**), and an attachable sterile pencil probe with a detection range up to 3.5 cm (**E**)

All surgical procedures are performed by an experienced oncology breast surgeon or an experienced surgical resident under direct supervision. At breast conserving surgery the surgeon localizes the RFID tag using the handheld, portable, battery-operated reader device (Fig. 2). The reader is bagged in a sterile drape for use in the sterile field. The reader has a loop probe with a detection range of up to 7 cm, and an attachable single-use sterile pencil probe (8 mm tip diameter) with a detection range up to 3.5 cm, intended for highly specific localization. The reader provides an audible signal that increases in pitch and volume when the probe is moved closer to the RFID tag. The reader also displays the distance from the probe to the tag in millimeters and the unique identification number of the RFID tag. The audible and visual feedback provide precise, real-time guidance during the excision procedure. Specimen radiography is performed to confirm successful retrieval of the RFID tag. Directly following the surgical procedure the surgeon completes a questionnaire on the duration and ease of the procedure.

**Fig. 3:**
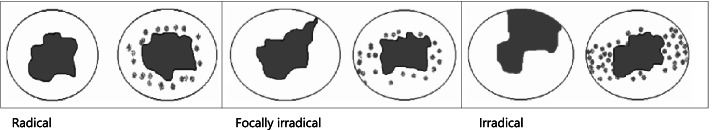
Classification of surgical margins [17]. Radical excision is defined as no tumor at inked cut edges in an adequately processed specimen. Focally irradical excision is defined as one limited area (≤ 4 mm) of tumor (invasive carcinoma and/or DCIS) at inked cut edges. Irradical excision is defined as a larger area (> 4 mm) of tumor or multiple areas of tumor at inked cut edges [17]

Histopathological analysis is performed following routine standard of care, including assessment of surgical margins. Surgical margins are classified as radical, focally irradical and irradical according to the Dutch breast cancer guideline [17] (Fig. 3). Irradical excision and indication for reexcision is recorded.

Any adverse events potentially related to the RFID localization procedure, noted by the involved study radiologist, surgeon, pathologist, or other health care providers, are recorded. Post-operative wound infections, noted during post-operative follow-up visits as usual, are collected from the medical records 30 days after surgery by a study team member.

After completion of inclusion all users of the RFID localization system are invited to complete a questionnaire on system usability using the validated 10-item System Usability Scale [18]. All data is collected using Castor Electronic Data Capture.

### Statistical analysis

Data will be analyzed using SPSS and RStudio. Descriptive statistical analysis will include the calculation of means, medians and interquartile ranges of the obtained data. The percentage of irradical excisions will be calculated by dividing the number of irradical excisions by the total number of excisions, with the corresponding 95% confidence interval (CI). The reexcision rate will be calculated by dividing the total number of reexcisions by the total number of excisions, with the corresponding 95% CI. Questionnaire data is evaluated using the five-level Likert scale.

### Sample size considerations

We aim to include a minimum of 200 patients over a study period of one year. This sample size is based on the primary study outcome of the percentage of irradical excisions and reexcision rate, which will be compared to standards of the National Breast Cancer Organisation Netherlands (NABON). The NABON standards set a maximum of irradical excisions of invasive carcinomas at 15% [19]. Based on earlier data we expect that with a study population of 200 women, approximately 174 will have invasive breast cancer [19]. To show that with RFID surgical guidance the upper boundary of the 95% CI of the percentage of radical excisions does not exceed the NABON standard of 15%, with 174 cases of invasive breast cancer, a maximum percentage of irradical excisions of 9% in our study population is needed. Therefore, a sample size of 200 women is considered feasible and sufficient to show a statistically and clinically relevant outcome.

## Discussion

The landscape of localization techniques is evolving fast in the search for the ideal method [4, 8–10]. Besides the RFID system, other novel, non-wire non-radioactive alternatives for lesion localization are under investigation, including SAVI SCOUT Radar localization and magnetic marker implantation (Magseed or MaMaLoc). Each technique comes with its own challenges.

The SAVI SCOUT system involves a 12-mm-long electromagnetic wave reflector that is activated by infrared light from a detector handpiece and a console that gives audible feedback to provide real-time guidance [20]. The magnetic resonance imaging (MRI) compatible radar reflector can be placed up to 5 cm in depth and up to 7 days before surgery using ultrasound or mammographic guidance. SAVI SCOUT is reported to be a reliable and effective alternative method but interaction with electrocautery can disrupt the signal or disable the reflector [21–23].

The Magseed system involves a 5-mm-long magnetic seed that can be placed up to 30 days preoperatively using ultrasound or mammographic guidance [24]. The Magnetic Marker Localization (MaMaLoc) is an experimental 3.5-mm-long magnetic marker [25]. The main challenge of using magnetic markers is the interference of metal surgical instruments with the detection of the marker, necessitating the use of special nonferromagnetic instruments. Magnetic marker localization is reported to be a reliable, effective and safe non-radioactive alternative to current localization techniques [24–27].

For both the SAVI scout and magnetic marker localization, as well as the RFID system, a downside is the need for two localization systems (two probes and two consoles) for breast conserving surgery requiring sentinel lymph node biopsy. This leads to a fuller operation field and requires the surgeon to switch between two techniques. Future registration for the use of novel localization methods in the axilla could resolve this. Further innovation should also focus on longer-term marker placement in patients undergoing neoadjuvant treatment. The current limited term that nonradioactive non-wire devices may be in place complicates its use in patients undergoing neoadjuvant treatment. In addition, the significant signal void artifacts on MRI from magnetic markers and RFID tags make response assessment on MRI virtually impossible (Supplemental material 1).

In conclusion, the field of localization techniques for nonpalpable breast lesions is moving fast and further research on novel approaches is essential to determine which are the localization techniques of the future. The RFID Localizer I trial will evaluate the feasibility of RFID surgical guidance for nonpalpable breast lesions in a prospective multicenter cohort study. Results may impact the future of localization techniques for nonpalpable breast cancer.

## Supplementary Information

**Additional file 1.**

## Data Availability

The datasets generated during the study will be available from the authors on reasonable request.

## Abbreviations

CC: Craniocaudal
CI: Confidence Interval
MLO: Mediolateral Oblique
MRI: Magnetic Resonance Imaging
NABON: Nationaal Borstkanker Overleg Nederland, National Breast Cancer Organisation Netherlands
RFID: Radiofrequency Identification
