# Supplementary material for: A multicenter prospective cohort study to evaluate feasibility of radio-frequency identification surgical guidance for nonpalpable breast lesions: design and rationale of the RFID Localizer 1 Trial
Source: BMC Cancer. 2022 Mar 22;22:305. doi: 10.1186/s12885-022-09394-7 (PMC8939217; doi:10.1186/s12885-022-09394-7)
Supplement: Supplementary file 1 — Additional file 1. [file 12885_2022_9394_MOESM1_ESM.docx]

**Supplemental material**


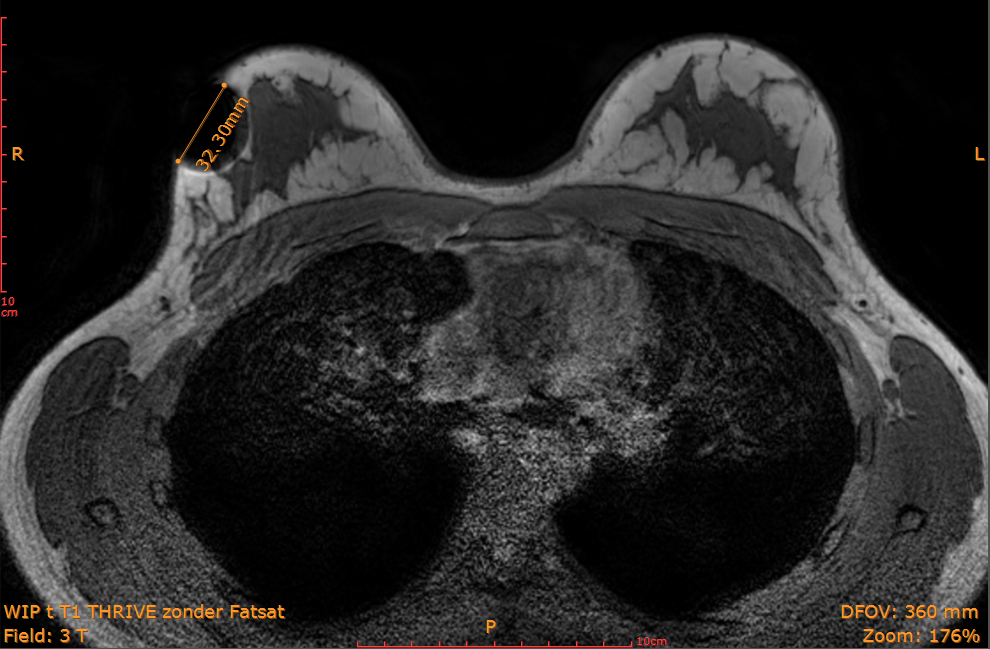


MR image (T1 THRIVE) obtained on 3.0 T unit of a test subject with an RFID tag taped to skin of the upper lateral quadrant of the right breast. Note signal void artifact of 3.2cm.
